# Supplementary figures and images for: Repeatability and reproducibility of a handheld quantitative G6PD diagnostic
Source: PLoS Negl Trop Dis. 2022 Feb 17;16(2):e0010174. doi: 10.1371/journal.pntd.0010174 (PMC8853557; doi:10.1371/journal.pntd.0010174)

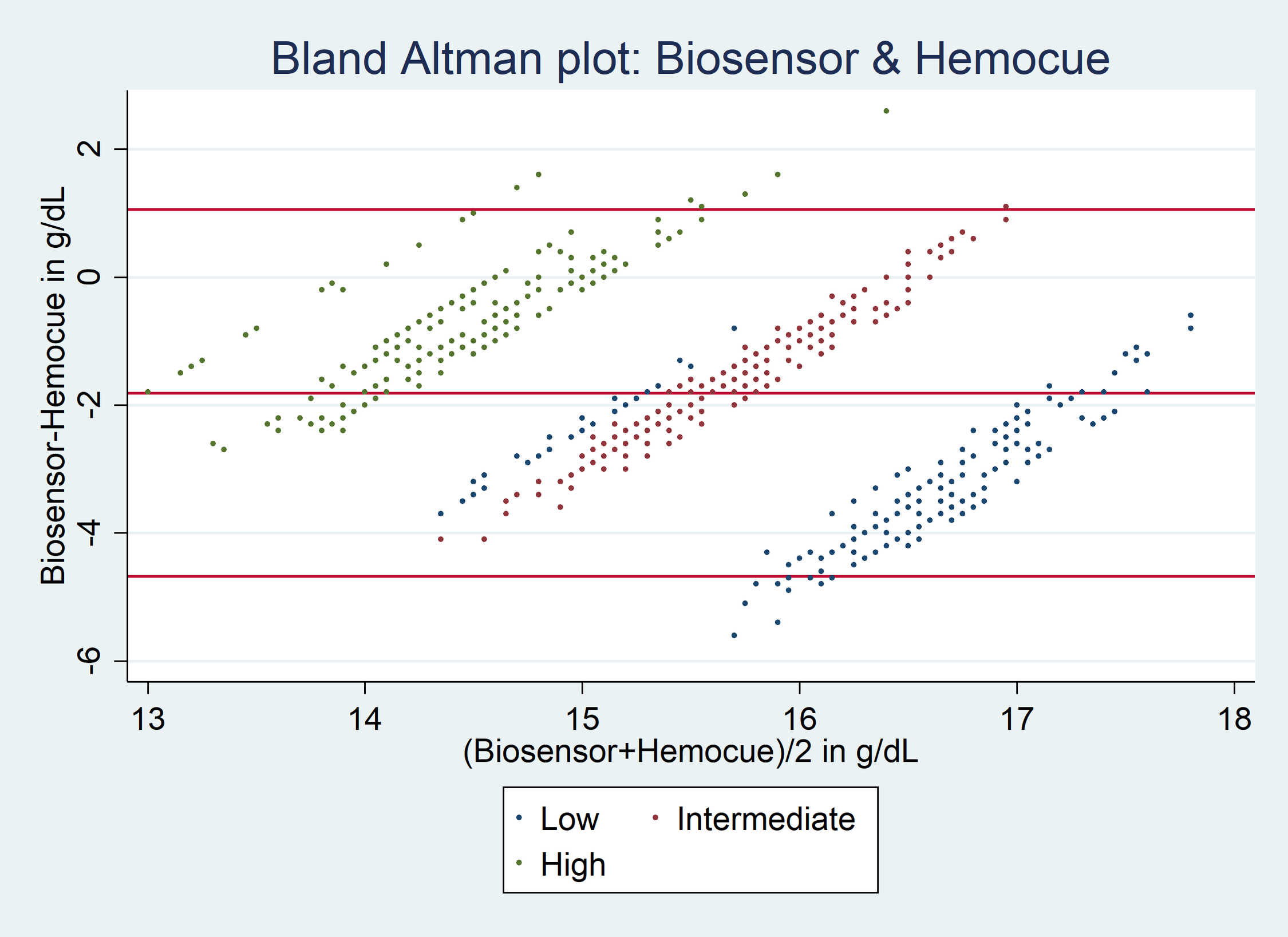

Supplement: S1 Fig — (TIF) [file pntd.0010174.s002.tif]

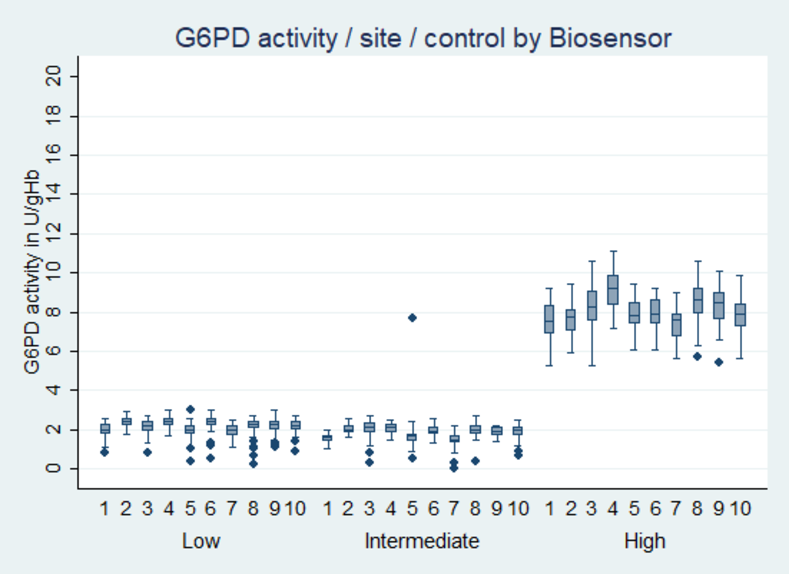

Supplement: S2 Fig — (TIF) [file pntd.0010174.s003.tif]

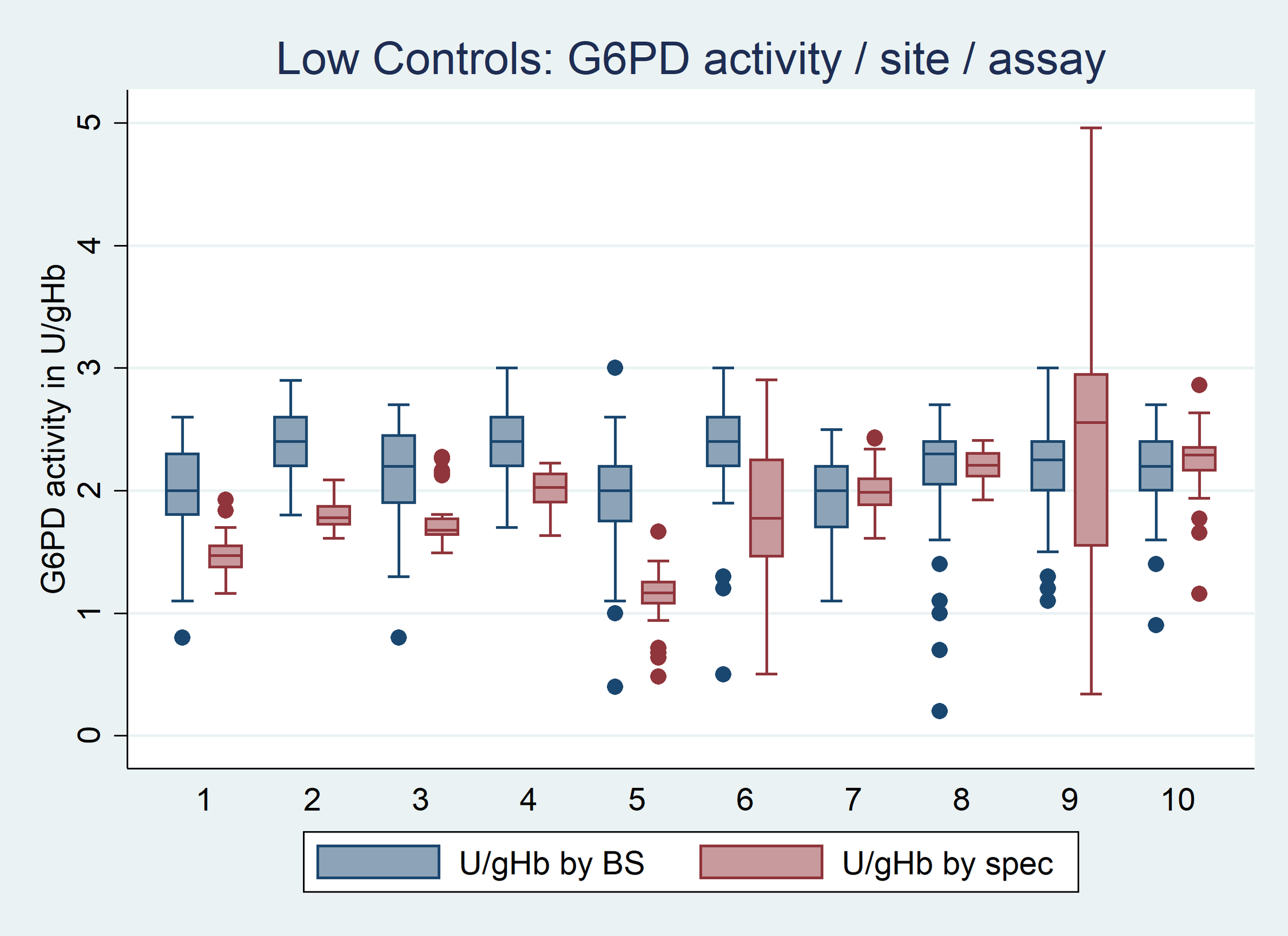

Supplement: S3 Fig — (TIF) [file pntd.0010174.s004.tif]

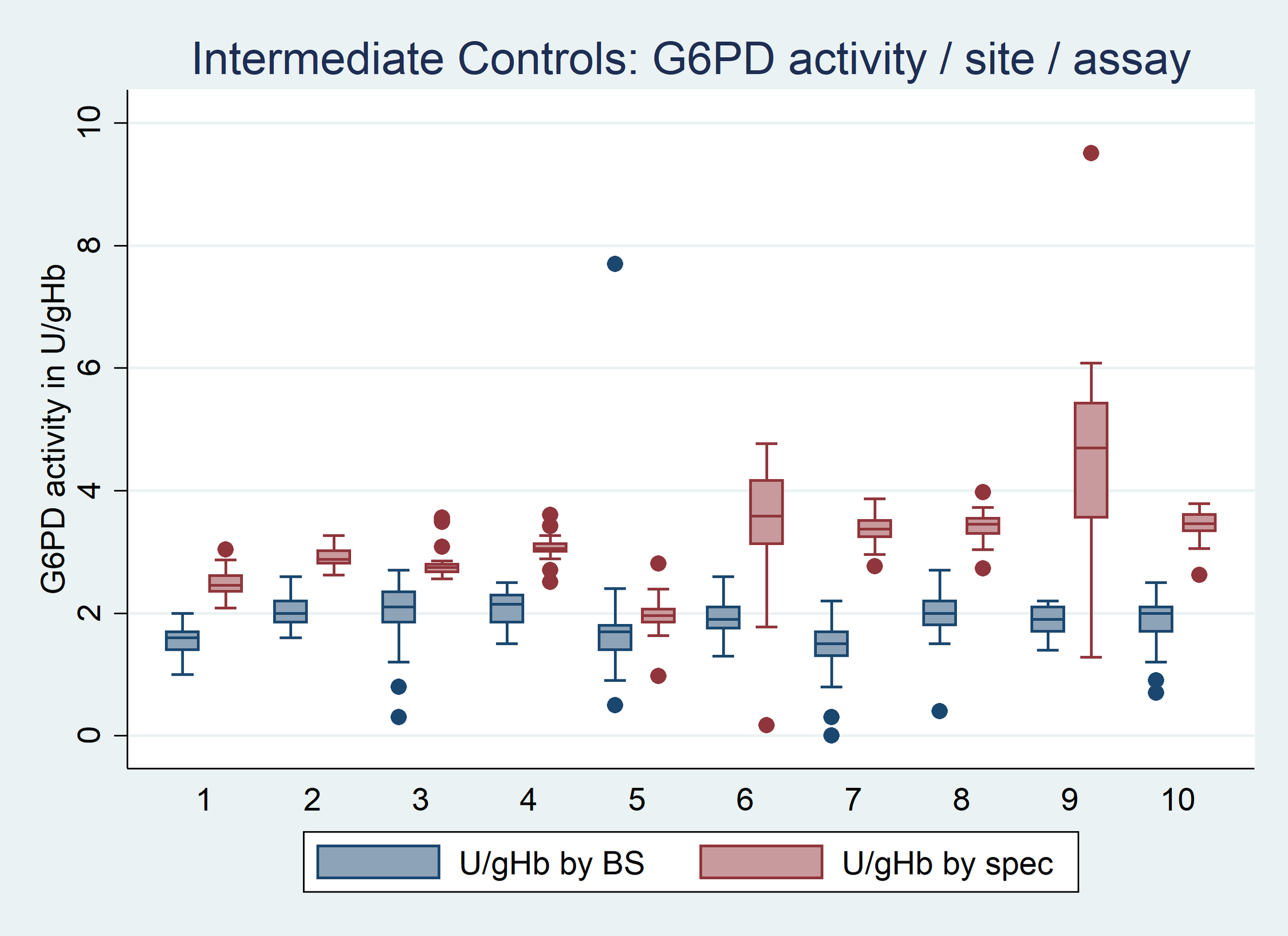

Supplement: S4 Fig — (TIF) [file pntd.0010174.s005.tif]

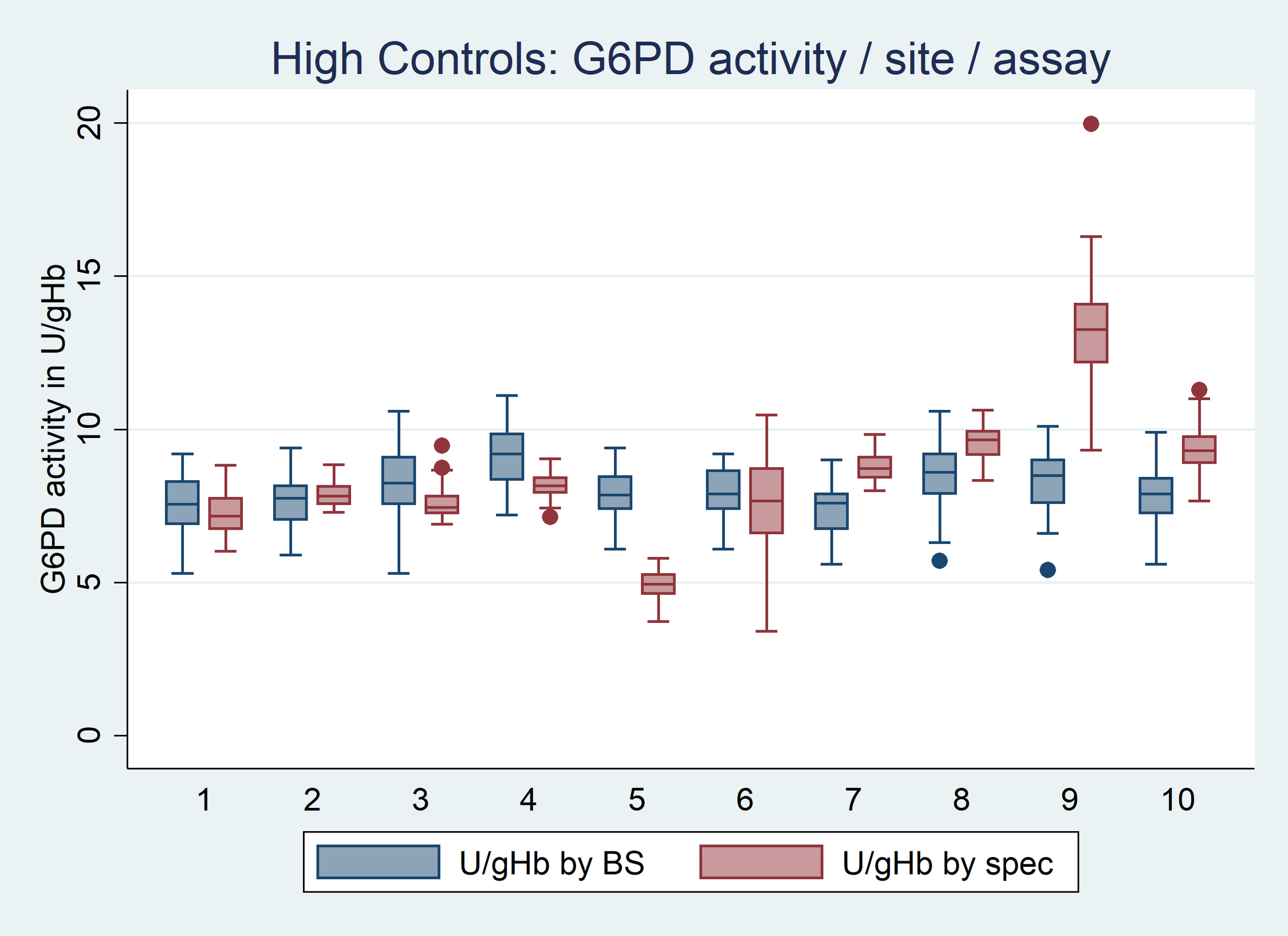

Supplement: S5 Fig — (TIF) [file pntd.0010174.s006.tif]

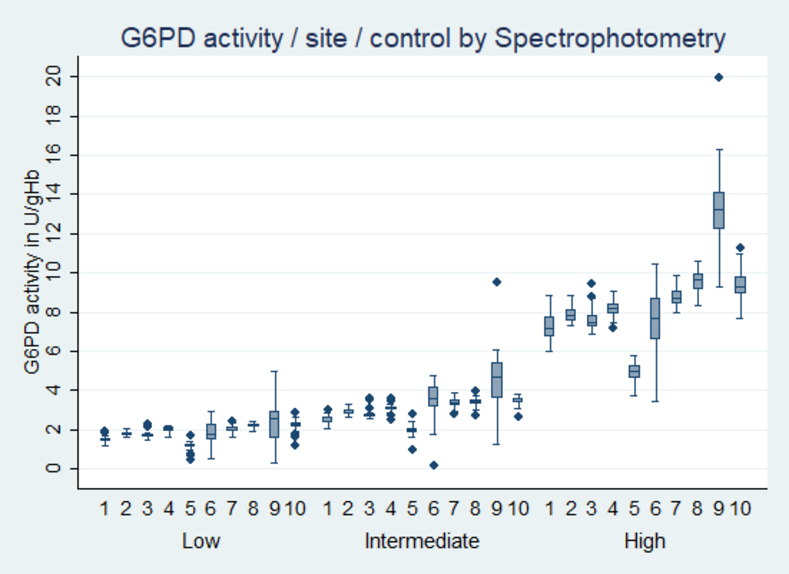

Supplement: S6 Fig — (TIF) [file pntd.0010174.s007.tif]
