# Supplementary material for: Repeatability and reproducibility of a handheld quantitative G6PD diagnostic
Source: PLoS Negl Trop Dis. 2022 Feb 17;16(2):e0010174. doi: 10.1371/journal.pntd.0010174 (PMC8853557; doi:10.1371/journal.pntd.0010174)
Supplement: S1 Table — (DOCX) [file pntd.0010174.s008.docx]

| **Site** | **Board** | **Approval number / waiver** |
| --- | --- | --- |
| Armed Forces Research Institute of Medical Sciences, Thailand | Walter Reed Army Institute of Research (WRAIR) Institutional Review Board | Waiver |
| Eijkmans Institute of Molecular Biology, Indonesia | Eijkman Institute Research Ethics Commission | EIREC #149 |
| Fundação de Medicina Tropical, Brazil | FMT-HVD Institutional Review Board | Waiver |
| George Mason University, USA | Office of Research Integrity and Assurance, Institutional Review Board | Waiver |
| Institut Pasteur, France | Institut Pasteur Institutional Review Board | Waiver |
| International Centre for Diarrheal Disease Research, Bangladesh | Institutional Ethical Review Committee (ERC) | Waiver |
| Menzies School of Health Research, Australia | Human Research Ethics Council of the Northern Territory | Waiver |
| PATH, USA | Office of research affairs and research determination committee | Non-human subjects research determination |
| Shoklo Malaria Research Unit, Mahidol-Oxford Tropical Medicine Research Unit, Faculty of Tropical Medicine, Mahidol University, Thailand | Ethics Committee of the Faculty of Tropical Medicine, Mahidol University and Oxford Tropical Research Ethics Committee | Waiver |
| Yale University, USA | Human Investigation Committee of Yale University | Waiver |
