# Supplementary material for: Repeatability and reproducibility of a handheld quantitative G6PD diagnostic
Source: PLoS Negl Trop Dis. 2022 Feb 17;16(2):e0010174. doi: 10.1371/journal.pntd.0010174 (PMC8853557; doi:10.1371/journal.pntd.0010174)
Supplement: S2 Table — (DOCX) [file pntd.0010174.s009.docx]

**S1 Table:** *lot numbers of ACS controls per Phase*

| **Control** | **Catalogue number** | **Lot number Phase A** | **Lot number Phase B** |
| --- | --- | --- | --- |
| **High** | HC-108 | I-91-10 | I-91-10 |
| **Intermediate** | HC-108IN | I-90-31 | I-90-31 |
| **Low** | HC-108DE | I-90-32* | I-91-32* |
| **Test strips** | 02G6S10 | G6S1020003 | G6S1020003 |

**lot numbers differed between Phase A and Phase B*
