# Supplementary material for: Repeatability and reproducibility of a handheld quantitative G6PD diagnostic
Source: PLoS Negl Trop Dis. 2022 Feb 17;16(2):e0010174. doi: 10.1371/journal.pntd.0010174 (PMC8853557; doi:10.1371/journal.pntd.0010174)
Supplement: S3 Table — (DOCX) [file pntd.0010174.s010.docx]

**S2 Table:** *coefficient of variation and median G6PD activity / Hb concentration measured per assay and device in Phase A*

|  | **Biosensor: G6PD activity in U/gHb** | | | **Biosensor: Hb in g/dL** | | |
| --- | --- | --- | --- | --- | --- | --- |
| **Device** | **Low CV, median (IQR, range)** | **Intermediate: CV, median (IQR, range)** | **High: CV, median (IQR, range)** | **Low: CV, median (IQR, range)** | **Intermediate: CV, median (IQR, range)** | **High: CV, median (IQR, range)** |
| **1** | 0.215, 0.7 (0.6 - 0.7, 0.3 - 0.8) | 0.169, 1.7 (1.5 - 1.8, 1.1 - 2.1) | 0.136, 7.7 (6.5 - 8.2, 6.1 - 9.6) | 0.046, 14.9 (14.6 - 15.5, 13.9 - 16.7) | 0.052, 14.8 (14.3 - 15.6, 14.1 - 16.6) | 0.060, 14.1 (13.5 - 14.6, 12.6 - 15.6) |
| **2** | 0.263, 0.8 (0.7 - 0.9, 0.1 – 1.0) | 0.138, 1.8 (1.7 - 1.9, 1.4 - 2.4) | 0.103, 7.8 (7.2 - 8.3, 6.5 - 9.1) | 0.060, 15 (14.3 - 15.7, 13.6 - 16.9) | 0.052, 15.5 (14.7 - 16, 13.9 - 16.4) | 0.058, 14.9 (14.1 - 15.1, 13.3 - 16.4) |
| **3** | 0.178, 0.7 (0.6 - 0.7, 0.4 - 0.9) | 0.192, 1.7 (1.6 - 1.9, 0.9 - 2.2) | 0.116, 7.8 (7.2 - 8.3, 5.7 - 9.3) | 0.069, 14.3 (13.9 - 15.2, 13.2 - 17) | 0.065, 14.6 (13.7 - 15.3, 13.1 - 15.9) | 0.054, 13.7 (13 - 14.2, 12.7 - 15) |
| **4** | 0.215, 0.7 (0.6 - 0.7, 0.3 - 0.8) | 0.169, 1.7 (1.5 - 1.8, 1.1 - 2.1) | 0.136, 7.7 (6.5 - 8.2, 6.1 - 9.6) | 0.046, 14.9 (14.6 - 15.5, 13.9 - 16.7) | 0.052, 14.8 (14.3 - 15.6, 14.1 - 16.6) | 0.060, 14.1 (13.5 - 14.6, 12.6 - 15.6) |
| **5** | 0.187, 0.7 (0.6 - 0.8, 0.4 - 0.9) | 0.167, 1.6 (1.4 - 1.8, 1.2 – 2.0) | 0.089, 7.7 (7.1 - 8.1, 6.3 - 8.7) | 0.057, 15.7 (15.2 - 16.4, 14.6 - 17.5) | 0.052, 15.6 (15.1 - 16.3, 13.9 - 16.8) | 0.059, 15.3 (14.2 - 15.6, 13.7 - 16.7) |
| **6** | 0.250, 0.6 (0.4 - 0.7, 0.4 - 0.8) | 0.152, 1.6 (1.4 - 1.7, 1.0 – 2.0) | 0.102, 7.6 (7.4 - 7.9, 6.7 - 9.9) | 0.058, 15 (14.4 - 15.8, 14 - 17) | 0.063, 15.2 (15 - 16.3, 13.7 - 17.1) | 0.035, 14.5 (14.1 - 15, 13.8 - 15.3) |
| **7** | 0.215, 0.6 (0.5 - 0.7, 0.3 - 0.8) | 0.187, 1.6 (1.4 - 1.8, 1.1 - 1.9) | 0.102, 7.4 (6.8 - 7.5, 6.5 - 9.6) | 0.046, 15 (14.8 - 15.5, 13.9 - 16.5) | 0.052, 15 (14.6 - 15.7, 14 - 16.9) | 0.065, 15.1 (14.5 - 15.3, 12.5 - 16.1) |
| **8** | 0.322, 0.6 (0.5 - 0.8, 0.2 - 0.8) | 0.169, 1.6 (1.4 - 1.7, 0.9 - 1.9) | 0.109, 7.8 (7.2 - 8.2, 6.0 - 8.7) | 0.056, 14.6 (14.3 - 15.2, 13.5 - 16.2) | 0.052, 15.2 (14.3 - 15.4, 13.4 - 16.1) | 0.097, 13.9 (13.2 - 14.9, 12.4 - 17.7) |
| **9** | 0.346, 0.7 (0.6 - 0.7, 0.1 - 0.9) | 0.176, 1.8 (1.6 - 1.9, 1.0 - 2.1) | 0.133, 8 (7.4 - 8.9, 6.6 - 10.5) | 0.051, 14.5 (14.1 - 14.9, 13.2 - 16.3) | 0.058, 14.1 (13.6 - 14.5, 12.3 - 15.2) | 0.051, 13.8 (13.5 - 14.2, 12.4 - 15.2) |
| **10** | 0.239, 0.7 (0.5 - 0.8, 0.4 - 0.9) | 0.101, 1.7 (1.5 - 1.7, 1.2 - 1.8) | 0.114, 7.5 (6.9 - 8, 5.9 - 9.2) | 0.062, 14.9 (14.2 - 15.4, 13.7 - 16.9) | 0.061, 15 (14.6 - 15.8, 13.9 - 17.4) | 0.059, 14.5 (13.9 - 14.9, 12.5 - 15.6) |
| **Pooled** | **0.260, 0.7 (0.6 – 0.8, 0.1 – 1.0** | **0.172, 1.7 (1.5 – 1.8, 0.6 – 2.4)** | **0.111, 7.6 (7.2 – 8.2, 5.7 – 10.5)** | **0.061, 14.8 (14.3 – 15.6, 12.9 – 17.5)** | **0.068, 14.9 (14.2 – 15.5, 12.3 – 17.5)** | **0.069, 14.1 (13.5 – 15.0, 12.0 – 17.7)** |
