# Supplementary material for: Repeatability and reproducibility of a handheld quantitative G6PD diagnostic
Source: PLoS Negl Trop Dis. 2022 Feb 17;16(2):e0010174. doi: 10.1371/journal.pntd.0010174 (PMC8853557; doi:10.1371/journal.pntd.0010174)
Supplement: S4 Table — (DOCX) [file pntd.0010174.s011.docx]

*S3 Table: Summary of readings for Biosensor (G6PD activity and Hb), spectrophotometry, and Hemocue across 10 sites in Phase B*

|  | **Biosensor: G6PD activity in U/gHb** | | | **Spectrophotometry: G6PD activity in U/gHb** | | |
| --- | --- | --- | --- | --- | --- | --- |
| **Site** | **Low: median (IQR, range)** | **Intermediate: median (IQR, range)** | **High: median (IQR, range)** | **Low: median (IQR, range)** | **Intermediate: median (IQR, range)** | **High: median (IQR, range)** |
| 1 | 2.0 (1.8 – 2.3, 0.8 – 2.6) | 1.6 (1.4 - 1.7, 1.0 – 2.0) | 7.6 (6.9 - 8.3, 5.3 - 9.2) | 1.5 (1.4 - 1.6, 1.2 - 1.9) | 2.5 (2.3 - 2.6, 2.1 - 3) | 7.2 (6.7 - 7.8, 6.0 - 8.8) |
| 2 | 2.4 (2.2 – 2.6, 1.8 – 2.9) | 2.0 (1.9 - 2.2, 1.6 - 2.6) | 7.8 (7.1 - 8.2, 5.9 - 9.4) | 1.8 (1.7 - 1.9, 1.6 - 2.1) | 2.9 (2.8 - 3, 2.6 - 3.3) | 7.8 (7.6 - 8.1, 7.3 - 8.9) |
| 3 | 2.2 (1.9 – 2.5, 0.8 – 2.7) | 2.1 (1.9 - 2.4, 0.3 - 2.7) | 8.3 (7.6 - 9.1, 5.3 - 10.6) | 1.7 (1.6 - 1.7, 1.5 - 2.1) | 2.7 (2.7 - 2.8, 2.6 - 3.1) | 7.5 (7.3 - 7.8, 6.9 – 8.2) |
| 4 | 2.4 (2.2 – 2.6, 1.7 – 3.0) | 2.2 (1.9 - 2.3, 1.5 - 2.5) | 9.2 (8.4 - 9.9, 7.2 - 11.1) | 2.0 (1.9 - 2.1, 1.6 - 2.2) | 3.1 (3 - 3.1, 2.5 - 3.6) | 8.2 (7.9 - 8.4, 7.1 – 9.0) |
| 5 | 2.0 (1.8-2.2, 0.4-3.0) | 1.7 (1.4-1.8, 0.5-7.7) | (7.9 (7.4-8.5, 6.1-9.4) | 1.2 (1.1-1.3, 0.5-1.7) | 2.0 (1.8-2.1, 1.0-2.8) | 4.9 (4.6-5.3, 3.7-5.8) |
| 6 | 2.4 (2.2-2.6, 0.5-3.0) | 1.9 (1.8-2.1, 1.3-2.6) | 7.9 (7.4-8.7, 6.1-9.2) | 1.8 (1.5-2.3, 0.5-2.9) | 3.6 (3.1-4.2, 0.2-4.8) | 7.7 (6.6-8.7, 3.4-10.5) |
| 7 | 2.0 (1.7 – 2.2, 1.1 – 2.5) | 1.5 (1.3 - 1.7, 0.0 - 2.2) | 7.6 (6.8 - 7.9, 5.6 - 9) | 2.0 (1.9 - 2.1, 1.6 - 2.4) | 3.4 (3.2 - 3.5, 2.8 - 3.9) | 8.7 (8.4 - 9.1, 8.0 - 9.8) |
| 8 | 2.3 (2.1 – 2.4, 0.2 – 2.7) | 2.0 (1.8 – 2.2, 0.4 – 2.7) | 8.6 (7.9 – 9.2, 5.7 – 10.6) | 2.2 (2.1 – 2.3, 1.9 – 2.4) | 3.4 (3.3 – 3.5, 2.7 – 4.0) | 9.7 (9.2 – 9.9, 8.3 – 10.6) |
| 9 | 2.3 (2.0 – 2.4, 1.1 – 2.5) | 1.9 (1.7 - 2.1, 1.4 - 2.2) | 8.5 (7.6 - 9, 5.4 - 10.1) | 2.6 (1.6 - 2.9, 0.3 - 5) | 4.7 (3.6 - 5.4, 1.3 - 9.5) | 13.3 (12.2 - 14.1, 9.3 – 20.0) |
| 10 | 2.2 (2.0 – 2.4, 0.9 – 2.7) | 2.0 (1.7 – 2.1, 0.7 – 2.5) | 0.125, 7.9 (7.3 – 8.4, 5.6 – 9.9) | 2.3 (2.2 – 2.4, 1.2 – 2.9) | 3.5 (3.3 – 3.6, 2.6 – 3.8) | 9.3 (8.9 – 9.8, 7.7 – 11.3) |
| **Pooled** | **2.2 (2.0-2.4, 0.2-3.0)** | **1.9 (1.6-2.1, 0.0-7.7)** | **8.0 (7.4-8.7, 5.3-11.1)** | **1.9 (1.6-2.2, 0.3-5.0)** | **3.1 (2.7-3.5, 0.2-9.5)** | **8.2 (7.4-9.2, 3.4-20.0)** |
| **Lot-specific ACS range** | 0.8-3.8 | 3.7-6.8 | 10.3-19.1 | 0.8-3.8 | 3.7-6.8 | 10.3-19.1 |
|  | **Biosensor: Hb in g/dL** | | | **Hemocue: Hb in g/dL** | | |
| 1 | 14.0 (13.4 - 14.3, 12.3 - 16.1) | 14.2 (13.6 - 14.8, 12.2 - 16) | 13.6 (12.9 - 14.2, 12 - 15.5) | 16.3 (16.0 - 16.4, 15.5 - 16.6) | 16.6 (16.5 - 16.7, 16.3 - 16.9) | 14.9 (14.3 – 15.0, 13.8 - 15.1) |
| 2 | 13.7 (13.4 - 14, 12.7 - 14.8) | 14.1 (13.7 - 14.3, 13.1 - 15.3) | 14.1 (13.7 - 14.5, 13.2 - 15.3) | 15.9 (15.7 - 16.4, 15.2 - 16.9) | 16.9 (16.7 - 17.1, 16.0 - 17.7) | 14.8 (14.6 - 15.1, 14.4 - 15.7) |
| 3 | 12.9 (12.7 - 13.5, 11.8 - 14.6) | 13.5 (13.2 - 13.7, 12.5 - 14.7) | 12.9 (12.5 - 13.3, 11.8 - 14.1) | 16.6 (16.0 - 16.8, 12.1 - 17.2) | 17.1 (16.9 - 17.2, 12.9 - 17.4) | 15.1 (14.5 - 15.4, 12.1 - 15.7) |
| 4 | 13.3 (12.8 - 13.7, 11.4 - 16.5) | 13.2 (13 - 13.7, 12.3 - 15.6) | 12.8 (12.5 - 13.1, 11.3 – 15.0) | 16.2 (15.8 - 16.7, 15.3 - 17.3) | 16.4 (16.3 - 16.7, 16.0 - 17.2) | 14.3 (14.1 - 14.8, 13.7 - 15) |
| 5 | 13.8(13.3-14.3, 12.2-14.7) | 14.0 (13.7-14.6, 12.7-16.0) | 13.1 (12.9-13.8, 12.0-14.6) | 13.3 (13.0-13.4, 11.3-14.1) | 13.4 (13.3-13.5, 12.1-14.0) | 12.8 (12.7-12.9, 11.5-13.3) |
| 6 | 12.9 (12.5-13.5, 12.1-15.7) | 13.3 (12.8-13.8, 12.0-15.1) | 12.7 (12.3-13.4, 10.9-14.3) | 15.7 (14.8-16.9, 13.8-20.5) | 15.1 (14.1-17.3, 13.4-19.0) | 15.5 (14.7-16.2, 13.4-17.9) |
| 7 | 12.8 (12.5 - 13.4, 9.9 - 15.5) | 13.3 (12.9 - 13.8, 11.6 - 15.1) | 12.9 (12.3 - 13.5, 10.9 - 14.7) | 15.1 (14.8 – 15.3, 14.6 – 15.8) | 15.9 (15.8 – 16.1, 15.2 – 16.7) | 13.4 (13.2 – 13.8, 13.0 – 14.1) |
| 8 | 13.1 (12.9 – 13.6, 12.1 – 15.9) | 13.8 (13.4 – 14.1, 12.6 – 15.3) | 12.9 (12.6 – 13.3, 11.8 – 14.9) | 13.1 (13.0 - 13.2, 12.9 - 13.4) | 13.5 (13.4 - 13.6, 13.2 - 13.7) | 12.9 (12.8 – 13.0, 12.6 - 13.2) |
| 9 | 13.5 (12.8 - 14.6, 11.1 - 18.2) | 13.9 (13.1 - 14.6, 11.7 - 19.1) | 13.4 (12.6 – 15.0, 10.3 - 20.7) | 13.3 (13.1 - 13.5, 13.0 – 14.0) | 13.5 (13.4 - 13.7, 13.1 - 13.9) | 13.2 (13.0 - 13.3, 12.8 - 13.5) |
| 10 | 13.5 (13.2 – 14.1, 12.2 – 14.7) | 13.9 (13.5 – 14.5, 12.7 – 15.8) | 13.7 (13.3 – 14.0, 12.5 – 15.7) | 15.0 (14.8 – 15.3, 14.4 – 15.7) | 15.7 (15.5 – 15.8, 14.5 – 16.2) | 13.5 (13.3 – 13.8, 13.0 – 14.3) |
| **Pooled** | **13.4 (12.9-13.9, 9.9-18.2)** | **13.7 (13.2-14.2, 11.6-19.1)** | **13.2 (12.7-13.7, 10.3-20.7)** | **15.3 (13.4-16.2, 11.3-20.5)** | **15.9 (13.6-16.7, 12.1-19.0)** | **14.0 (13.1-14.8, 11.5-17.9)** |
| **Lot-specific ACS range** | 13.0 | 13.2 | 13.1 | 13.0 | 13.2 | 13.1 |
