# Supplementary material for: Repeatability and reproducibility of a handheld quantitative G6PD diagnostic
Source: PLoS Negl Trop Dis. 2022 Feb 17;16(2):e0010174. doi: 10.1371/journal.pntd.0010174 (PMC8853557; doi:10.1371/journal.pntd.0010174)
Supplement: S5 Table — (DOCX) [file pntd.0010174.s012.docx]

*S4 Table. Phase B coefficient of variation (CV) of G6PD measurements by Biosensor vs Spectrophotometry and Haemoglobin measurements by Biosensor vs Hemocue*

|  | **G6PD: Biosensor and Spectrophotometry repeatability** | | | | | | | | |
| --- | --- | --- | --- | --- | --- | --- | --- | --- | --- |
| **Site** | **Low** | | | **Intermediate** | | | **High** | | |
|  | **Biosensor CV** | **Spec. CV** | **p** | **Biosensor CV** | **Spec. CV** | **p** | **Biosensor CV** | **Spec. CV** | **p** |
| 1 | 0.210 | 0.103 | <0.001 | 0.165 | 0.079 | <0.001 | 0.125 | 0.094 | 0.016 |
| 2 | 0.108 | 0.064 | <0.001 | 0.128 | 0.052 | <0.001 | 0.104 | 0.052 | <0.001 |
| 3 | 0.140 | 0.108 | <0.001 | 0.248 | 0.093 | <0.001 | 0.125 | 0.068 | <0.001 |
| 4 | 0.117 | 0.069 | <0.001 | 0.141 | 0.059 | <0.001 | 0.111 | 0.050 | <0.001 |
| 5 | 0.254 | 0.192 | 0.019 | 0.589 | 0.138 | <0.001 | 0.103 | 0.092 | 0.302 |
| 6 | 0.244 | 0.291 | 0.152 | 0.119 | 0.274 | <0.001 | 0.108 | 0.185 | <0.001 |
| 7 | 0.169 | 0.090 | <0.001 | 0.277 | 0.062 | <0.001 | 0.107 | 0.050 | <0.001 |
| 8 | 0.261 | 0.058 | <0.001 | 0.195 | 0.065 | <0.001 | 0.119 | 0.064 | <0.001 |
| 9 | 0.198 | 0.431 | <0.001 | 0.139 | 0.310 | <0.001 | 0.124 | 0.137 | 0.391 |
| 10 | 0.163 | 0.122 | 0.013 | 0.231 | 0.062 | <0.001 | 0.125 | 0.086 | 0.001 |
| **Pooled** | **0.210** | **0.282** | **<0.001** | **0.270** | **0.274** | **0.643** | **0.129** | **0.259** | **<0.001** |
|  | **Hb: Biosensor and Hemocue repeatability** | | | | | | | | |
|  | **Biosensor CV** | **Hemocue CV** | **p** | **Biosensor CV** | **Hemocue CV** | **p** | **Biosensor CV** | **Hemocue CV** | **p** |
| 1 | 0.053 | 0.019 | <0.001 | 0.056 | 0.010 | <0.001 | 0.064 | 0.028 | <0.001 |
| 2 | 0.034 | 0.028 | 0.076 | 0.034 | 0.020 | <0.001 | 0.037 | 0.023 | <0.001 |
| 3 | 0.047 | 0.077 | <0.001 | 0.036 | 0.073 | <0.001 | 0.044 | 0.054 | 0.066 |
| 4 | 0.072 | 0.036 | <0.001 | 0.050 | 0.017 | <0.001 | 0.056 | 0.026 | <0.001 |
| 5 | 0.050 | 0.035 | 0.001 | 0.049 | 0.027 | <0.001 | 0.052 | 0.026 | <0.001 |
| 6 | 0.062 | 0.100 | <0.001 | 0.062 | 0.107 | <0.001 | 0.061 | 0.072 | 0.140 |
| 7 | 0.073 | 0.019 | <0.001 | 0.058 | 0.019 | <0.001 | 0.057 | 0.026 | <0.001 |
| 8 | 0.052 | 0.009 | <0.001 | 0.043 | 0.009 | <0.001 | 0.048 | 0.011 | <0.001 |
| 9 | 0.134 | 0.018 | <0.001 | 0.124 | 0.015 | <0.001 | 0.158 | 0.015 | <0.001 |
| 10 | 0.047 | 0.024 | <0.001 | 0.056 | 0.032 | <0.001 | 0.042 | 0.028 | <0.001 |
| **Pooled** | **0.072** | **0.097** | **<0.001** | **0.066** | **0.099** | **<0.001** | **0.079** | **0.075** | **0.126** |

*Legend: Spec = spectrophotometry, CV = coefficient of variation*
