# Supplementary material for: Repeatability and reproducibility of a handheld quantitative G6PD diagnostic
Source: PLoS Negl Trop Dis. 2022 Feb 17;16(2):e0010174. doi: 10.1371/journal.pntd.0010174 (PMC8853557; doi:10.1371/journal.pntd.0010174)
Supplement: Attachment — Submitted filename: Ley_B-et al_Reponse to reviewers_v1.0.docx [file pntd.0010174.s013.docx]

**Author responses to reviewer feedback:**

**We thank the Editor and three Reviewers for their detailed review of our manuscript and appreciate their positive feedback and opportunity for providing clarifications and further details. Please see below (in bold) a detailed response to the Reviewers’ questions.**

**LINE NUMBERS REFER TO THE CLEAN RESUBMITTED VERSION OF THE MANUSCRIPT**

**Methods**

**Reviewer #1:** This manuscript reports on and intra-laboratory and inter-laboratory analysis of G6PD assays carried out by a commercial device called BIOSENSOR.

The results show that, overall, the agreement among multiple BIOSENSOR devices, is good, but with exceptions. The comparison among several distant laboratory must have posed considerable challenges and it is an enterprise for which the Authors are to be commended.

However, I have to raise several general criticisms.

1. The main motive for this study, as stated in the introduction and elsewhere, is to avoid hemolytic complications from primaquine or tafenoquine. The assessment of G6PDd in males is very easy: it does not require either a spectrophotometric assay or BIOSENSOR, as it can be done by the fluorescent spot test or by other inexpensive screening tests. The need for a quantitative tests regards only females. Surprisingly, this is not stated anywhere.

**We agree individuals above and below 30% can be identified by qualitative diagnostics. However, short course primaquine and the recently introduced tafenoquine may require higher cut-offs and these can only be addressed by a quantitative diagnostic to date. We have clarified this in the abstract where it now reads: “*The introduction of short course, high dose primaquine and single dose tafenoquine for the radical cure of Plasmodium vivax present unique opportunities for more effective control of the parasite. Roll out will require a reliable quantitative point-of-care diagnostic that can identify glucose-6-phosphate dehydrogenase deficient individuals at risk of drug-induced haemolysis, especially females.* “ (lines 34 to 37).**

2. In view of the above, in order to assess BiOSENSOR the focus should be on the 'intermediate' group: specifically, on the success rate of the device in detecting heterozygotes with a range of G6PD activities. Unfortunately, by using a single intermediate control the work was not designed to assess this success rate. In addition, it is precisely in the intermediate group that problems were encountered.

**There are already published data on the accuracy of the Biosensor in subjects with different G6PD phenotypes, including intermediate females (cited in the Introduction). The study was designed to assess precision, not accuracy. For clarification we have added the following sentence to the introduction: “*The aim of this study was not to assess accuracy, but to determine the Biosensor’s repeatability (assay precision when repeated under constant conditions) and reproducibility (assay precision under different conditions, such as across devices, operators and sites), since robust performance of these characteristics is necessary for rolling-out universal Biosensor thresholds for clinical decisions*.” (lines 128 to 132)**

3. The Authors should briefly explain how the BIOSENSOR works. Is G6PD measured by NADPH production, or by formazan production, or in what other way? Is Hb measured by cyanmethemoglobin production or in what other way?

**We have expanded the respective section in the introduction, by adding the following: “*The test strip contains 5-bromo-4-chloro-3-indolyl-phosphate (BCIP) that is reduced to violet nitro blue tetrazolium (NBT) in the presence of G6PD enzyme, the colour intensity is directly proportional to G6PD activity and is measured through reflectance photometry.*** ***The Biosensor device quantifies Hb concentration using a photo-reflectance based algorithm informed by the sample’s colour intensity.*** ***This is measured on a separate spot to that for the G6PD activity.*” (lines 116 to 120)**

4. Lyophilization is not a standard way to process blood samples for a G6PD assay (and it may be a factor in some of the problems encountered). Given the design of their study, the Authors should show what was the agreement between G6PD values obtained in one lab on a set of samples, fresh versus after lyophilisation and re-constitution; and on 0 to 48 hours after re-constitution.

**Since G6PD activity in fresh blood samples declines rapidly over time we had to revert to commercial lyophilized blood samples when sending out standardized controls to ensure that all laboratories were using identical samples. We agree that the lyophilised samples may not be directly comparable to the original fresh blood samples, but we do not think this has had an impact on repeatability and reproducibility of the device. This is stated in the limitations section of the discussion: “*G6PD activity and Hb levels were measured in commercial lyophilized controls to ensure standardisation, while the Biosensor and reference assays are developed for testing fresh venous or capillary blood. Stabilizing agents contained in the controls may have affected the Biosensor and/or reference method and this effect may differ by assay.*” (lines 393 to 396).**

**The proposed equivalence experiment would be of great interest, however, is outside the scope of this project.**

5. In heterozygous (intermediate) females what matters from the point of view of hemolysis is, rather than the level of G6PD activity in a hemolysate, the proportion of G6PDd red cells within their red cell mosaicism. Of course the two do correlate, but somewhere in the paper this important fact should be mentioned.

**We have added the following paragraph to the introduction: “*The G6PD gene is located on the X-chromosome, accordingly males are either hemizygous deficient or normal, whereas females are homozygous deficient, normal, or heterozygous for the gene. Heterozygous females have two distinct RBC populations, and G6PD activity of heterozygous females – and their associated haemolytic risk – is dependent on the ratio of both populations, defined through the random process of lyonization*” (lines 81 to 86)**

**Reviewer #2:** The overall study design is appropriate, allowing direct comparison between sites. However, as the authors also mentioned, the devices used in this study (Biosensor and Hemocue) all require the use of fresh blood. So the use of lyophilised RBCs may not faithfully reflect the real situations.

**Kindly see comment 4 of reviewer 1**

**Reviewer #3:** The objectives of the study are very clearly articulated, the design is appropriate, the sample set and size are clearly described and justified, the statistical analysis is robust and there are no ethical or regulatory concerns that I can tell.

There are no major additional analyses/experiments required.

**Many thanks for the review**

--------------------

**Results**

**Reviewer #1:** Line 145. It would be important to know exactly what these controls are: I could not find any G6PD controls on the ACS site. What was the original G6PD activity on the fresh blood sample of each? I think Here and throughout the manuscript I assume 'high' means a sample from a G6PD normal person: why use the term high?

**We have added the following additional information in lines 151 to 161:** **“*To ensure that identical samples were tested across all sites, commercial controls were used, with all controls within one phase being from the same lot (Analytical Control Systems, Inc., Indiana, USA; S1 Table). ACS controls are routinely used to monitor quality of reference G6PD testing by spectrophotometry. They are derived from whole blood obtained from human donors in FDA licensed centers and pooled to represent high, intermediate, or low G6PD activity (Cat. Nos.: HC-108, HC-108IN, and HC-108DE respectively). ACS provide lot specific G6PD activity range guides and Hb estimates. These are based on automated spectrophotometry estimates conducted by Pointe-Scientific on the reconstituted controls (32 vials in total). ACS recommend that laboratories develop their own in-house ranges. As a reference, the manufacturer guides are presented in the Results, but these are guides only given the potential differences in spectrophotometry protocols followed (ACS personal communication)****”.* **The intermediate samples are a mixture of high and low activity blood designed to provide an intermediate sample between high and low, not necessarily obtained from heterozygous donors. The original G6PD activity levels on the fresh blood were not available from the manufacturer. The three categories of ACS controls provide a range of G6PD enzyme activity levels allowing the technical characteristics of the G6PD assays to be measured. The aims of our study were not clinical investigations of activity levels across different G6PD genotypes.**

**The suggested ranges for each control are presented in figures 2, 5, and 6 as blue, red and green shade and are also included in table 2 for Phase A, in table S3 for phase B, and in lines 299 to 307 again for phase B: “*Biosensors and controls were shipped to one laboratory in Bangladesh, Brazil, France and Indonesia, two laboratories in Thailand, and four in the USA for further testing. Staff at six laboratories had previous experience with the Biosensor. Pooled readings from Low controls were within the recommended range (0.8U/gHb – 3.8U/gHb) by Biosensor (median: 2.2U/gHb, IQR: 2.0 – 2.4) and spectrophotometry (1.9U/gHb, IQR: 1.6 – 2.2). However Intermediate pooled readings by Biosensor (1.9U/gHb, IQR: 1.6 – 2.1) and spectrophotometry (3.1U/gHb, IQR: 2.7 – 3.5) were below the manufacturer recommended range (3.7U/gHb – 6.8U/gHB), as were the readings of High controls (Biosensor: 8.0U/gHb, IQR: 7.4 – 8.7; spectrophotometry: 8.2U/gHb, IQR: 7.4 – 9.2; recommended range 10.3U/gHb – 19.1 U/gHb).”***

Fig. 2. In this figure there are several strange features on which there is no comment in the text until the discussion, and the legend is incomplete: are all the cricles outliers? Shaded areas are said to be the recommended ranges (presumably recommended by ACS). I could not find what were the original G6PD activity values of the control samples provided; but even the spectrophotometric assay gives values way below recommended ranges in the intermediate and 'high' areas. This suggests that the lyophilization creates a problem. The red dotted line is said to be the median of the low controls, but I think it is the median of the intermediate controls. Similar apparent mistakes in defining the horizontal lines are replicated in the captions of Figs. 5 and 6.

**We apologise, there was a typo in the legends of figures 2, 4, and 5 referring only to Low controls, we have corrected this throughout and have added the manufacturer of the controls to each legend to clarify who provided the recommended ranges. The legends now read: “*Legend: Dots represent outliers, Blue shade = recommended range for ACS Low controls, Red shade = recommended range for ACS Intermediate controls, Green shade = recommended range for ACS High controls, Blue dotted line = median activity of ACS Low controls across all devices, Red dotted line = median activity of ACS Intermediate controls across all devices, Green dotted line = median activity of ACS High controls across all devices.*” (figures 2, 4, and 5)**

Fig. 3. In the Hb measurement the agreement among devices is clearly not very good: whatever the overall statistics say, most of the data from device number 5 (from the left) are above most of the data from device number 4. Since I expect Hb to be independent of G6PD, I don't understand why the reference (Hemocue) is way higher than BIOSENSORs in low and intermediate, and about right in 'high'.

**We did not comment on this observation due to the sample size of three. We have highlighted the limitations of assessing Hb values from lyophilized samples in the discussion, where it says: *“Determining accuracy of Biosensor Hb readings against the Hemocue reference assay with reconstituted lyophilized controls is of limited clinical relevance*.” (lines 386 to 388). We also report findings from previous field studies that were designed to assess accuracy in Biosensor Hb measurements: “*A study from the US compared paired Hb measurements from fresh, venous, samples by Biosensor and Hemocue (model 201+) and found the mean difference to be 1.0g/dL (22); a study comparing Biosensor Hb readings from venous blood samples to the results of a complete blood count (CBC), found readings to differ by 0.4g/dL (21), and a recent study from Brazil found the mean difference again to be less than 1 g/dl*.” (lines 388 to 392)**

Fig. 4. The overall correlation between BIOENSOR and spectrophotometry is obviously OK; but the fact remains that in the intermediate group the bulk is about 3 IU/G Hb by spectrophotometry, and it is instead about 2 by BIOSENSOR. The superiority of spectrophotometry is supported by Suppl figure S4.

**We have clarified this by adding the following sentence to the conclusion: “*The characteristics of the Biosensor demonstrated in this study, and the good performance reported from field studies (21, 22), indicate that the Biosensor could be a valuable quantitative point-of-care diagnostic; however we found that spectrophotometry, when performed well, remains the gold standard with precision superior to the Biosensor.”* (lines 413 to 416)**

Fig. 5. In phase B, by the Authors' own admission, the low values of the 'high' controls and the lack of discrimination between low and intermediate controls are a bit of a disaster.

**This is highlighted in the discussion: “*In Phase B the Biosensor did not distinguish between Low and Intermediate controls. In fact, the results were significantly lower for Intermediate compared to Low controls and this was consistent across all sites; in contrast spectrophotometry in Phase B was able to distinguish between all three control categories.”* (lines 376 to 379) and again in the conclusion where we state: “*****Clinical data will be important to further investigate the poor discriminatory power of the Biosensor at low G6PD activities observed here with the lyophilised samples*.” (lines 420 to 422)**

Fig. 6. In spite of considerable scattering of values among different labs, the results are clearly better by spectrophotometry than by BIOSENSOR.

**Kindly see comment Fig.4 of reviewer 1**

**Reviewer #2:** Yes.

**Reviewer #3:** The results are clearly and completely presented and the supplementary data adds to the manuscript.

The figures and tables are of sufficient quality and clarity, I would however suggest to combine figures 2 & 3 and figures 5 & 6 in a panel figures so as to make direct comparison easier for the reader.

In addition, I would revise the tables and condense the information into fewer tables/add some of the tables to the supplementary data.

**We have combined figures 5 and 6 and tables 3 and 4. We are concerned that the readability of Figs 2 and 3 may be compromised by merging these together. Given that Fig 2 concerns G6PD results and Fig 3 is Hb, we believe these are sufficiently distinct to retain as individual figures.**

--------------------

**Conclusions**

**Reviewer #1**: Overall, the Authors have given a reasonably critical account of the shortcomings of BIOSENSOR emerging from their study. The emphasis on reproducibility, related to precision, cannot overcome the rather glaring problem with accuracy.

**Aim of this study was to assess precision, not accuracy. This is stated in the abstract, where it reads: “*The device can only be considered for routine care if repeatability and reproducibility of the assay are high”* (lines 40 to 41). To clarify this further we have added the following sentence in the introduction: “*The aim of this study was not to asses accuracy again, but to determine the Biosensor’s repeatability (assay precision when repeated under constant conditions) and reproducibility (assay precision under different conditions, such as across devices, operators and sites), since robust performance of these characteristics is a necessary basis to rolling-out universal Biosensor thresholds for clinical decisions“* (lines 128 to 132)**

The statement on lines 388-393 suggests that using lyophilized material was a weak point in the design of the study.

**We agree, and this is reflected in the limitations section of the discussion, where it says:** “***Our findings have several limitations. G6PD activity and Hb levels were measured in commercial lyophilised controls to ensure standardisation, while the Biosensor and reference assays are developed for testing fresh venous or capillary blood.”* (lines 393 to 395).**

**We would also like to note that this presents a significant challenge in conducting performance evaluations across multiple laboratories. Shipment of fresh or frozen blood across multiple laboratories in multiple countries is not reliable for an enzyme assay interlaboratory reproducibility and repeatability study. While lyophilized samples are not ideal, they are the currently only available controls.**

Line 414 onwards. It is a bit of an anti-climax that, having conducted a deliberate study on agreement among multiple sites, the authors have to quote previous studies on the reliability of BIOSENSOR; and they then proceed to state that their work needs to be "confirmed in clinical settings", after it has been found wanting in experienced laboratory settings.

**To limit the possible sources of variation, we selected to first compare the Biosensor performance in controlled settings where performance could also be compared to the reference assay, spectrophotometry. The study does show a higher variability in precision compared to the spectrophotometer and Hemocue, as can perhaps be anticipated given the lower complexity of the test. The study also does show that unlike spectrophotometry, the Biosensor performance is independent of end-user experience with the test. The study establishes this important baseline, which can inform the evaluation and interpretation of the performance of this test in clinical settings.**

**Reviewer #2**: Yes.

**Reviewer #3**: The conclusions are supported by the data and the limitations of the analysis are described in some detail. The authors briefly touch on how the data can inform the topic under study and briefly touch on public health relevance.

**We would like to thank the reviewers for their comments and suggestions**

--------------------

**Summary and General Comments**

**Reviewer #1:**

**Questions are addressed in the Methods section on page 1 of this document.**

**Reviewer #2:** This study evaluated the repeatability and reproducibility of the quantitative G6PD device (SD Biosensor) using standard commercial controls with high, intermediate and low G6PD activities. The results showed high repeatability and reproducibility of the device in a multi-lab setting. However, the study showed significant variations of the results from spectrometry-based measurements, which the authors speculated may have resulted from the variations from Hemocue measurement of the hemoglobin (Hb) level. As the authors discussed, the major caveat is that both the SD Biosensor and Hemocue are designed for using fresh blood, whereas the study used lyophilized controls.

1. While the study design using the same lots of controls allows direct comparison of the performance of the SD Biosensor in different labs, all controls were non-anemic and had a similar level of Hb. In endemic conditions, many vivax patients are anemic with much lower levels of Hb. Given that the variations in results may derive from the Hb measurement, it would be appropriate if the measurement was also done using diluted controls to mimic anemic situation.

**This is a good point. Biosensor readings should currently not be considered if the measured Hb is ≤7g/dL, excluding severely anaemic patients. We have added this to the manuscript: “*The handheld device displays G6PD activity (in U/gHb) and haemoglobin (Hb) levels (in g/dL) two minutes after applying the blood buffer solution, however the manufacturer indicates that results cannot be considered if Hb readings are equal to or below 7g/dL*.” (lines 120 to 123). We have also changed the conclusion which now reads: “*Finally, it will be important to verify whether the consistent and robust performance demonstrated here is maintained when the device is operated under routine conditions and in anaemic patients, as well as to define training requirements for intended users at the point-of-care*.” (lines 422 to 425)**

2. Comparison between the SB biosensor and spectrometry results showed that the latter could differentiate the low from intermediate levels of G6PD activities, whereas SD biosensor could not. It is doubted whether the discrepancy indeed reflects the lower sensitivity of SD biosensor in discriminating low vs intermediate level activity or the standard controls tested in this study are not appropriate.

**Kindly see comment 4 of reviewer 1 above**

Minor comments

1. Need to synchronize spellings (Br or Am, but not both) - e.g., line 37, haemolysis; line 81, hemolysis; line 43, lyophilised; line 54, lyophilized

**We have changed and standardized the spelling throughout the manuscript.**

3. Line 214-217: please clarify whether the comparison was done for Low, Intermediate or High separately, or these reflect the combined results.

**These findings are combined for all three controls. We have clarified this: “… *Hb* *readings differed significantly (p<0.001, adjusted R^2^ = 0.121). Compared to device 1 (baseline), devices 3 and 9 had significantly lower Hb readings with a difference of 0.477 g/dL (p=0.007) and 0.623 g/dL (p<0.001) respectively, while device 5 had a significantly higher Hb result with a difference of 0.665 g/dL (p<0.001) when comparing readings from all three controls (Fig 3, S2 Table*).” (lines 227 to 231)**

4. Figure 2: specify the shared areas are recommended... for the Biosensor device. Also clarify what do the dots represent (individual measurement)?

**Dots represent outliers, we have added this as explanation to the legends for figures 2, 3 and 5. The legends now read: “*Dots represent outliers, Red circled bars = reference method (Hb normalized spectrophotometry result), Blue shade = recommended range for ACS Low controls, Red shade = recommended range for ACS Intermediate controls, Green shade = recommended range for ACS High controls, Blue dotted line = median activity of ACS Low controls across all devices excluding spectrophotometry, Red dotted line = median activity of ACS Intermediate controls across all devices excluding spectrophotometry, Green dotted line = median activity of ACS High controls across all devices excluding spectrophotometry*”.**

5. Line 223-224. Why do different dotted lines represent the same? I think red line shows intermediate controls, while green dotted line shows the high control. The same mistakes are also found in Fig 5 and Fig 6 legends.

**This was a typo and we have corrected this for figures 2, 3, and 5. Kindly see the above response for the updated text.**

6. Line 236: It shows that the normalized activity was positively correlated. Are they significantly different (for Fig 1, Intermediate, it looks like the SD biosensor readings and spectrometry results are quite different).

**We think this question refers to figure 2. We have added mean differences to the respective section that now reads: “*****The Hb-normalized G6PD activity readings of the Biosensor and spectrophotometry were positively correlated across all three control categories (rs = 0.859, p<0.001), however median readings differed significantly for Low (mean difference: -0.1 U/gHb, 95% limit of agreement [95%LoA]: -0.8 to 0.5, p<0.001) and Intermediate controls (mean difference: -1.2 U/gHb, 95%LoA: -2.3 to -0.1, p<0.001), while median activities did not differ significantly for High controls (mean difference: -0.1 U/gHb, 95%LoA: -2.3 to 2.1, p=0.554*).” (lines 251 to 256)**

6. For the supplementary tables, please include the statistical results in these tables too.

**We have added the pooled CV to S2 table and double checked that pooled results are included in all other tables as well.**

**Reviewer #3**: The authors present G6PD enzyme activity measurement comparisons by two different methods in their paper titled: The STANDARD G6PD test (SD Biosensor) shows good repeatability and reproducibility in a multi-laboratory comparison. The paper is very well written and the data analysis and presentation is clear.

**Many thanks for the review.**

Please find below some minor comments I would like to make:

1. Would the authors please elaborate in more detail on the differences in between the recommended range set by the manufacturer and the range found by Biosensor/spectrometry. How do the company determine G6PD activity ranges? Do the company give any more information with regards to the controls i.e. from male or female, SNPs in question, country of origin of controls?

**Kindly see our reply to the first comment of reviewer 1**

2. Would the authors please discuss in more detail the inability of Biosensor to distinguish in between low and intermediate G6PD activity, especially in phase B. It seems that the reason why there is so much overlap in phase B, is that the median low G6PD activity is higher and the median intermediate G6PD activity is similar to phase A. How, if at all, would this be influenced by different batches?

**We have clarified that this study was not designed to assess accuracy by adding the following sentence to the introduction: “*The aim of this study was not to asses accuracy...* “ (line 128) We state in the discussion section that: “*In Phase B the Biosensor did not distinguish between Low and Intermediate controls. In fact, the results were significantly lower for Intermediate compared to Low controls and this was consistent across all sites; in contrast spectrophotometry in Phase B was able to distinguish between all three control categories*.” (lines 376 to 379). We have also changed the conclusion which now reads: “*Clinical data will be important to further investigate the poor discriminatory power of the Biosensor at low and intermediate G6PD activities observed here with the lyophilised samples.”* (lines 420 – 422).**

**Unfortunately, batch numbers for Low controls differed between phase A and B and this does not allow us to compare Low findings from phase a with Low findings from phase B directly.**

3. Would the authors please discuss the potential relevance of the inability to distinguish in between low and intermediate G6PD activity? What will it mean in terms of giving PQ to people/deciding on cut-offs/dosage regimes etc.?

The authors only describe data acquisition on controls and in well-equipped laboratory settings. It will be highly interesting to see results from field studies using the Biosensor.

**The study was not designed to assess accuracy, but only precision. The observed phenomenon will require further research in clinical and field studies with fresh blood samples. To highlight this, we have rephrased the conclusion which now reads: “*Clinical data will be important to further investigate the poor discriminatory power of the Biosensor at low and intermediate G6PD activities observed here with the lyophilised samples.*” (lines 420 - 422).**

**Additional changes introduced:**

We have made minor corrections to wording throughout the manuscript, and have added reference 23 which has recently been published and is relevant to the manuscript:

Zobrist et al (2021): **Evaluation of a point-of-care diagnostic to identify glucose-6-phosphate dehydrogenase deficiency in Brazil,** *PLoS NTD***,** DOI: 10.1371/journal.pntd.0009649

We have amended the affiliations of one of the authors, Didier Menard.
