# Supplementary material for: Repeatability and reproducibility of a handheld quantitative G6PD diagnostic
Source: PLoS Negl Trop Dis. 2022 Feb 17;16(2):e0010174. doi: 10.1371/journal.pntd.0010174 (PMC8853557; doi:10.1371/journal.pntd.0010174)
Supplement: Attachment — Submitted filename: BREIN_Rebuttal2_v2.docx [file pntd.0010174.s014.docx]

**Author responses to reviewer feedback 2:**

**We thank the Editor and Reviewers for their detailed review of our manuscript. Please see below (in bold) a detailed response to the Reviewers’ questions.**

**LINE NUMBERS REFER TO THE CLEAN RESUBMITTED VERSION OF THE MANUSCRIPT**

**General criticism 1.** The Authors refuse to make the simple statement that the diagnosis of G6PD deficiency in males is easy and does not require a Biosensor. They prefer to say "especially females".

**We have revised the abstract that now reads: “*****While deficient males can be identified using a qualitative diagnostic test, the genetic make-up of females requires a quantitative measurement.*” (lines 40 to 41)**

**General criticism 2.** I understand that the purpose of the study was precision, because on accuracy 'there are already published data'. To me it is a reason of concern that, with accuracy already established, it is not confirmed in this work. It does not make sense to me that the need for a quantitative test is at the basis of promoting Biosensor, and then the Authors do not know the original (true) values of G6PD activity in the samples they have distributed: I find this unacceptable.

**We recognize the point made by the reviewer, but the mere fact that the ACS controls did not behave as intended even for the reference assay highlights the challenges for demonstrating accuracy for an enzyme assay, and it is the reason we limit the analysis in this study to precision. The study design, as mentioned by the reviewer, was complicated and required the use of samples that would be standardized across participating laboratories. We were not able to establish our own reference ranges but relied on the ranges provided by the control’s manufacturer. We have clarified this in the methods section: “*ACS recommend that laboratories develop their own in-house ranges, however, provides ranges per control category as well. We were unable to establish spectrophotometry-based ranges applicable to all sites due to the inherent site-specific variability of spectrophotometry (15), we therefore considered the manufacturer recommended ranges*.” (lines 167 – 171)**

**General criticism 3**. This clarification is appropriate: I think it is a good addition to the manuscript.

**Many thanks for the suggestion.**

**General criticism 4.** The Authors essentially seem to agree that lyophilization was not a good idea (as pointed out also by another Reviewer). In principle, one might question whether the repeatability assessed on lyophilized samples would be valid for fresh samples. This is buttressed by the Authors' own statement that "Determining accuracy of Biosensor Hb readings against the Hemocue reference assay with reconstituted lyophilized controls is of limited clinical relevance." This puts into question the entire Hb data, that are half of the data in the paper.

**The quoted sentence refers to accuracy of Hb readings and we agree with the reviewer that accuracy can’t be estimated from our study design. This is emphasized by the following sentence in the limitations section of the discussion: “*Our findings have several limitations. G6PD activity and Hb levels were measured in commercial lyophilised controls to ensure cross-laboratory standardisation, but the Biosensor and reference assays are developed for testing fresh venous or capillary blood. Stabilizing agents contained in the controls may have affected the Biosensor and/or reference method and this effect may differ by assay. Repeatability and reproducibility of each assay should not have been impacted by this difference, but it may have affected accuracy of either device which is best assessed using fresh blood samples (21, 22)*. “ (lines 403 – 409)**

**Following the reviewer’s suggestions, we have further clarified the limitations of using lyophilized samples by adding the following sentence at the beginning of the methods: “*Despite the Biosensor and reference method spectrophotometry being developed for fresh blood we had to use lyophilized and reconstituted standardized controls instead in order to ensure identical samples were used throughout the study period (25)*.” (lines 141 – 143)**

**General criticism 5.** The added sentence is not well phrased. The G6PD activity measured in a hemolysate depends on the ratio between the two cell populations; the potential severity of hemolysis depends on the proportion of G6PD deficient red cells, because these are the ones susceptible to hemolysis from primaquine or tafenoquine.

**We agree and have changed the respective sentence in the introduction, which now reads:** *“Heterozygous females have two distinct red blood cell (RBC) populations, G6PD normal and G6PD deficient, that circulate in a ratio determined through the random process of lyonization (1). Therefore, the G6PD activity levels of heterozygous females – and their associated hemolytic risk - is dependent on the proportion of deficient cells, those cells at greatest risk of drug induced hemolysis”* **(lines 88 – 92)**

**Additional comments.**

I was interested and not surprised that the "ACS recommend that laboratories develop their own in-house ranges". This has either not been done, or the data are not shown: in my view this should be mentioned in the discussion as a limitation. In addition, I still think that readers may be confused by the fact that activity of normal samples is called "high". As for intermediate activity, I can only reiterate that since this is the important range, it is unfortunate that only one set of samples was used within this range.

**We could not establish our own reference ranges in the course of this study and have clarified this as follows: “*ACS recommend that laboratories develop their own in-house ranges but provides guideline ranges per control category as well. We were unable to establish spectrophotometry-based ranges applicable to all sites due to the inherent site-specific variability of spectrophotometry, we therefore considered the manufacturer recommended ranges*” (lines 167 -171).**

**Having demonstrated the Biosensor’s good reproducibility we could have established reference ranges based on the Biosensor but believe that this would have been invalid and circular.**

**As suggested by the reviewer we have changed the discussion: “*While providing reference ranges, the manufacturer ACS suggests developing in-house reference ranges for all controls. We were unable to establish spectrophotometry-based ranges applicable to all sites due to the inherent site-specific variability of spectrophotometry (15) and instead considered the ranges provided. This approach likely explains why G6PD readings generated by Biosensor and spectrophotometry were below the ACS manufacturer’s recommended range and Hb readings by either assay did not match the guideline point estimates. This was consistent across different devices when assessed by a single user and by different laboratories. Unfortunately, the supplier was unable to provide additional controls from the same lots for further testing to clarify this issue. The observed narrow activity ranges meant that there was little difference in activity levels between the Intermediate and Low controls, limiting the activity range that was assessed.*” (lines 409 - 418)**

**Given the unknown impact the lyophilization process has on G6PD activity we aimed to disassociate the samples from their clinical equivalents and hence defined control categories as “Low”, “Intermediate” and “High”.**

**The answer to my criticism of Fig. 5 is unsatisfactory**. The Authors now say that "Clinical data will be important to further investigate the poor discriminatory power of the Biosensor at low G6PD activities": how on earth will "clinical data" help, when the device has failed in highly qualified labs?

**We tested a total of three distinct and standardized lyophilised controls and found that readings from two of these controls overlapped by Biosensor. We don’t think that drawing conclusions from this small sample size on the discriminatory power of the Biosensor is valid. We have clarified this in the conclusion where it now says: “*The precision of the Biosensor demonstrated in this study, and the good accuracy reported from field and other evaluation studies (21-23, 27), …”* (lines 426 – 427). To highlight the discrepancy observed between the three samples tested in this study and former studies designed to assess accuracy we had previously already written: “*Clinical data will be important to further investigate the poor discriminatory power of the Biosensor at low and intermediate G6PD activities observed with the lyophilised samples*.” (lines 433 – 435).**

**This is also reflected in the changed abstract, which now reads: “*Repeatability and inter-laboratory reproducibility of the Biosensor are more consistent compared to spectrophotometry, but the device did not reliably discriminate between intermediate and low G6PD activities of the lyophilized specimens. Clinical studies are now required to assess the performance of the test in practice*.” (lines 60-62)**

**Reviewer #1: Considering all of the above**, and the many limitations of this study, some of which are admitted by the Authors, I find the claim of 'consistent and robust performance' (line 428) seriously inconsistent.

**We have changed the conclusion which now reads: “*The precision of the Biosensor demonstrated in this study, and the good accuracy reported from field and other evaluation studies (21-23,27), indicate that the Biosensor could be a valuable quantitative point-of-care diagnostic; however, we found that spectrophotometry, when performed well, remains the gold standard with precision superior to the Biosensor.”* (lines 426 – 429).**

**We have replaced “consistent and robust performance” in the sentence cited above to precision: “*Finally, it will be important to verify whether the observed precision demonstrated here is maintained when the device is operated under routine conditions and in anaemic patients, as well as to define training requirements for intended users at the point-of-care.*” (lines 435 – 438)**

**Reviewer #1: In summary**, there are many unsatisfactory results in the performance of Biosensor in this study, with respect to both Hb and G6PD activity. The whole point of a quantitative test versus a qualitative test ought to be to detect heterozygotes at risk, and the device has proven wanting precisely in this respect. The Authors correctly recognize that Biosensor is inferior to the standard spectrophotometric assay. **None of these serious problems is reflected in the Abstract**, that gives instead the impression that everything is OK. The abstract should state instead that there are problems with the Biosensor and that the device needs to be improved.

**We have revised the abstract and have rephrased the conclusion as follows: “*Repeatability and inter-laboratory reproducibility of the Biosensor were more consistent compared to spectrophotometry, but the device did not reliably discriminate between intermediate and low G6PD activities of the lyophilized specimens. Clinical studies are now required to assess the devices performance in practice.*” (lines 60 – 63).**

**We would like to reiterate that the accuracy of the STANDARD test has already been assessed across multiple geographies including Brazil, Bangladesh, Thailand, UK and US showing the discriminatory power of the test for deficient and intermediate activities on clinical specimens (Alam et al, 2018, Pal et al, 2019, Zobrist et al, 2021, Pal et al, 2021). While those indicate clinical utility of the test, this current study describes the underlying precision.**

**We have also revised the abstract to meet the 300 words limit and have added an additional article as reference that has been published since the first submission:**

27.	Pal S, Myburgh J, Bansil P, Hann A, Robertson L, Gerth-Guyette E, et al. Reference and point-of-care testing for G6PD deficiency: Blood disorder interference, contrived specimens, and fingerstick equivalence and precision. PloS one. 2021;16(9):e0257560.1.	Luzzatto L, Ally M, Notaro R. Glucose-6-Phosphate Dehydrogenase Deficiency. Blood. 2020.
