# Supplementary material for: Repeatability and reproducibility of a handheld quantitative G6PD diagnostic
Source: PLoS Negl Trop Dis. 2022 Feb 17;16(2):e0010174. doi: 10.1371/journal.pntd.0010174 (PMC8853557; doi:10.1371/journal.pntd.0010174)
Supplement: Attachment — Submitted filename: BREIN_rebuttal3.docx [file pntd.0010174.s015.docx]

**Author responses to reviewer feedback 3:**

**We thank the Editor for the detailed review of our manuscript. Please see below a detailed response to all raised comments and concerns.**

**LINE NUMBERS REFER TO THE CLEAN RESUBMITTED VERSION OF THE MANUSCRIPT**

*1.	The title should be revised to describe the study theme and design. It is not necessary to declare that which may arguably mislead one to perceive proven suitability for purpose. This study does not do that. Something like; “Repeatability and reproducibility of a portable quantitative G6PD test device within and among users and laboratories”*

We have changed the title to: “**Repeatability and reproducibility of a handheld quantitative G6PD diagnostic”**

*2.	In the abstract, line 60, the authors seem to infer inferiority of the spectrophotometric standard compared to the POC device. This is unnecessary. Simply express that good repeatability and inter-laboratory reproducibility occurred, but the device did not reliably discriminate low and intermediate samples.*

We have changed the respective sentence that now reads: “**Repeatability and inter-laboratory reproducibility of the Biosensor were good, though the device did not reliably discriminate between intermediate and low G6PD activities of the lyophilized specimens**.” (lines 59 – 60).

*The argument for set global cut-off values guiding 8-aminoquinoline treatment decisions (line 78 & elsewhere) may not be supported by observations limited to relatively very few specimens, none of which represented fresh peripheral blood.*

We have changed the respective section which now reads: “**If further studies under real life conditions generate comparable results, the Biosensor will allow global cut offs for G6PD deficiency to be defined and will greatly simplify the roll out of novel highly effective radical cure treatment regimens for *P. vivax* infections**.” (lines 76 – 78).

We have changed lines 428 – 431, where it now says: “**The reproducibility observed in this study indicates that the technology is likely to permit direct comparison of results generated by different Biosensor devices and trained users ... If confirmed in clinical settings, the Biosensor has the potential to be an important tool to facilitate the broader roll out of 8-aminoquinoline radical cure**” The statement highlights the potential the Biosensor bears if our and former observations are confirmed under real life conditions, so would like to keep this statement as is.

3.	*If the technicians involved were not trained and certified as competent/capable in the spectrophotometric assay (as they were with the Biosensor), this should perhaps be expressed. If that is so, you may wish to express less firmly that statement at line 374-6.*

We have clarified this in line 201 – 203, where it now says: “**All technicians conducting the experiments had at least a bachelor’s degree or higher, several years’ experience of working in a laboratory and familiarity with spectrophotometry, however not necessarily with the G6PD assay used in this study**”.

In lines 373-376 it says: “**In contrast there was significant variation when G6PD activity was measured by spectrophotometry between sites despite standardized controls and procedures, a phenomenon that has been reported previously**” and we would like to keep this statement as is; this observation agrees with findings from a previous meta-analysis earlier (Pfeffer et al, 2020).

4.	*The authors have failed to make an important point (in my opinion). The intended use of the instrument is to generate a number that informs a “go” vs. “no go” decision on 8-aminoquinoline administration. That number is 70-80% of normal G6PD activity (GSK or WHO), below which fall all “intermediate” and “deficient” phenotypes. The lack of discernment of “low” and “intermediate” by the Biosensor is thus of no practical consequence. You see my opinion on this diametrically opposes that of the Reviewer. Separating “low” and “intermediate” is certainly critical for scientific study of the phenomenon and solving the clinical problem, but in the hands of the intended end-user there is no need to separate them – both safely get the “no go” on treatment. It is a single number, greater than, or less than – the only precision of importance is in that classification.*

We would like to thank the editor for this important point, which we have added to the discussion: “**Clinical data will be important to further investigate the poor discriminatory power of the Biosensor at low and intermediate G6PD activities observed with the lyophilised samples, however given that the Biosensors’ most probable designation will be to distinguish G6PD normal individuals from those with less than normal activity (at a cut-off of 70% activity for Tafenoquine) the observed poor discriminatory power at lower activities is unlikely to be of significant practical relevance**” (lines 432 – 436)

5.	*Your concluding statement (line 438-40) exceeds the limitations imposed by your experimental approach. Your work convincingly demonstrates reproducibility and repeatability of the Biosensor. Your work certainly does not prove the instrument “offers reliable quantitative diagnosis of G6PD status” – how could you possibly prove that with this design? Your evidence, in fact, seems to argue the opposite conclusion. You may articulate that the findings are consistent with an expectation that the instrument may consistently inform a safe treatment decision based on locally relevant thresholds for that decision.*

We have changed the concluding sections as follows: “**In conclusion, our findings suggest that the Biosensor offers reproducible quantitative diagnosis of G6PD status at the point-of-care in the hands of well-trained technicians. If repeatability and reproducibility as well as the previously reported accuracy are confirmed under real life conditions, the biosensor has the potential to simplify access to effective radical cure of P. vivax malaria**” (lines 439 – 443)
